# Does the unified protocol really change personality more than other interventions? Probably little if at all: a commentary on a recently-published study

**DOI:** 10.3389/fpsyt.2023.1280905

**Published:** 2023-11-09

**Authors:** Lorenzo Lorenzo-Luaces

**Affiliations:** Department of Psychological and Brain Sciences, Indiana University, Bloomington, IL, United States

**Keywords:** depression, personality, CBT (cognitive-behavioral therapy), transdiagnostic cognitive behavior treatment (TCBT), Unified Protocol (UP)

The Unified Protocol (UP) is a manualized cognitive-behavioral therapy (CBT) intended to be transdiagnostic. UP-CBT is meant to be transdiagnostic not just in the sense that it can used with patients who have multiple diagnoses, but in the sense that it is meant to target vulnerabilities that are shared across internalizing disorders (1). UP-CBT is hypothesized to alter the personality trait neuroticism, the tendency to feel intense negative emotions in response to stress. The idea that UP-CBT targets neuroticism has as its strongest support a reanalysis of a randomized controlled trial (RCT) by Barlow et al. (2). In the reanalysis by Sauer-Zavala et al. (3), changes in self-reported neuroticism were greater in UP-CBT than in a waiting list control or in “single-disorder” CBT. If UP-CBT reduces vulnerability to internalizing symptoms more than other CBTs, this would be a huge discovery as targeting hypothesized mechanisms of psychopathology may improve outcomes above and beyond targeting symptoms [(1), but see (4)].

Osma et al. (5) recently published the results of an RCT in which individuals with an emotional disorder, were randomized to group-based UP-CBT or treatment as usual (TAU). TAU consisted of pharmacotherapy or psychotherapy described as “non-protocolized CBT,” administered according to clinical judgment and availability. With a sample of 488, the study is powered to detect small-medium differences between conditions.

As with all research, there are minor things to quibble about (e.g., no accounting for therapist effects, no correction for multiple comparisons, no accounting for the clustered nature of the group data). I take issue with something more major: how the results were presented, and, therefore, how they may be interpreted. There is some inconsistency in how the findings regarding personality change are presented, leaving open the possibility for a reader to misinterpret the findings.

The authors structure their results section by first discussing changes over time within each of the treatment conditions. They present “uncontrolled effect sizes” which characterize the magnitude of change by comparing scores at a follow-up period with scores at baseline *within each treatment condition*. Within-treatment changes were generally large and appeared larger in UP-CBT than in TAU. The authors add another section where they discuss time-by-condition interactions. In an RCT like this one, a time-by-condition interaction indicates whether outcomes differed over time between conditions and is usually the test of interest. At *p* < 0.05, there were statistically significant time-by-condition interactions in predicting depression, anxiety, and quality of life (see Table III; *d*s = 0.16–0.20, all *p*s < 0.05). Time-by-condition interactions were *not* statistically significant (i.e., all *p*s > 0.05) for neuroticism (d = 0.09), negative affect (d = 0.11), extraversion (d = 0.14), and positive affect (d = 0.06). Thus, relative to TAU, UP-CBT produces very small changes in measures of personality that are not statistically significant.

The authors accurately summarize the between-condition effect sizes by writing that “both interventions produced comparable changes in neuroticism, negative affect, extraversion, and positive affect.” The abstract is somewhat equivocal, mentioning that extraversion does not improve with TAU, which a reader could interpret to mean it does with UP-CBT [but, see (6)]. In the discussion, the authors go on to write:

“UP *produced large reductions in neuroticism* and negative affect, which is again consistent with previous literature … These findings support the idea that the UP is a useful intervention to address emotional dysregulation (high neuroticism), a mechanism believed to be shared by all patients with [internalizing disorders].” (emphasis added)

I think this way of presenting the findings, presenting the uncontrolled effect sizes in the results, mentioning that extraversion does not improve with TAU, and in the discussion saying that UP-CBT produced large changes in neuroticism, has the potential to be misleading. In terms of the personality outcomes, the findings are a lack of statistically-significant differences between UP-CBT and TAU (i.e., *p*s > 0.05), a lack of *clinically-significant* differences (i.e., low between-group effect sizes), and a failure to replicate prior findings. The largest difference found here in personality/temperament was the difference in extraversion (*d* = 0.14) but this is not statistically significant (*p* = 0.08). While a *p* of 0.08 may seem like an interesting trend, it is one of many analyses reported. To put it in context, the study had at least 9 outcomes and at least 3 post-treatment periods leading to at least 27 tests and they are reported by treatment condition and as a between-condition comparison so there's over 60 tests.

Uncontrolled effect sizes are problematic because it is unclear what amount of change is caused by the treatment and which is caused by threats to internal validity like natural recovery, maturation, regression to the mean, or other processes (7). I worry readers will be left with the impression that the “UP produced large reductions in neuroticism and negative affect” when in reality very little of that (*d* = 0.06–0.14) seems attributable to UP-CBT *per se*. I am not arguing that uncontrolled effect sizes should *never* be reported. For example, it is helpful to know that suicidal ideation decreases in brief interventions, even if that decrease is not more than in active controls (8). But, any claim about the specific effects of UP-CBT require proof that UP-CBT has such specific effects.

Using Comprehensive Meta-analysis, I synthesized the neuroticism results from the Osma et al. (5) and Zauer-Savala et al. (3) studies as these are two RCTs comparing UP-CBT to another condition in changing personality. The results suggested that, relative to other CBTs (i.e., single-disorder CBT, TAU-CBT), UP-CBT was associated with decreases in neuroticism that are small (*d* = −0.14, 95% CI: −0.30, 0.01) and not statistically significant at *p* < 0.05. I suspect these results would be even smaller accounting for allegiance effects and publication bias (9). So, this new information from the trial by Osma et al. does not invalidate the study by Sauer-Zavala et al. (3) but instead suggests that UP-CBT has small effects on neuroticism beyond the moderate changes that already occur in other forms of CBT (10). However, none of my critiques dampen my enthusiasm for something the article does quite well. It highlights that the public health burden of internalizing disorder is so great that we need to rethink current models of care to put a spotlight on interventions that can be effective and highly scalable like transdiagnostic group-based CBT. I commend the authors for such an interesting study.

## Author contributions

LL-L: Conceptualization, Formal analysis, Investigation, Methodology, Project administration, Software, Supervision, Validation, Writing—original draft, Writing—review & editing.
